# Fetal cardiac tumors: prenatal diagnosis, management and prognosis in 18 cases

**DOI:** 10.4274/jtgga.galenos.2020.2019.0180

**Published:** 2020-12-04

**Authors:** Mustafa Behram, Süleyman Cemil Oğlak, Züat Acar, Salim Sezer, Helen Bornaun, Aytül Çorbacıoğlu, İsmail Özdemir

**Affiliations:** 1 Clinic of Perinatology, University of Health Sciences Turkey, Kanuni Sultan Süleyman Training and Research Hospital, İstanbul, Turkey; 2 Clinic of Obstetrics and Gynecology, University of Health Sciences Turkey, Gazi Yaşargil Training and Research Hospital, Diyarbakır, Turkey; 3 Clinic of Pediatric Cardiology, University of Health Sciences Turkey, Kanuni Sultan Süleyman Training and Research Hospital, İstanbul, Turkey

**Keywords:** Fetal cardiac tumors, rhabdomyoma, tuberous sclerosis

## Abstract

**Objective:**

To evaluate the long-term follow-up of patients with fetal cardiac tumors (FCTs), and to review the literature regarding advances in diagnosis and management of FCTs in the last decade.

**Material and Methods:**

In this retrospective study, pregnant women referred to a single center maternal-fetal medicine unit between 2013 and 2018 for advanced ultrasonography, were reviewed. Pediatric cardiology counseling was offered to women whose fetuses had FCTs. All patients were evaluated according to revised diagnostic criteria for tuberous sclerosis complex (TSC). Medical treatment was administered to patients with FCTs ≥30 mm or if they were symptomatic. Everolimus therapy at a dose of 2x0.25 mg twice a week for three months was started in the postnatal period.

**Results:**

Out of the 75,312 patients referred 18 (0.024%) were diagnosed with FCTs. Six were referred with fetal arrhythmias and the others were diagnosed with FCTs during routine follow-up. Ten patients (55%) with FCTs were diagnosed with TSC. All tumors were assessed to be rhabdomyoma. Mean tumor diameter in fetuses with TSC was significantly larger than those without TSC (29.8±14.1 mm versus 9.3±4.8 mm, respectively; p=0.004). All patients (n=2) who received medical therapy had a diagnosis of TSC and multiple FCTs and a reduction in tumor size occurred. Tumor size decreased in eight patients spontaneously during follow-up, but increased in one patient who had multiple locations but no TCS. No change in size was observed in the remaining seven cases. None of the fetuses died during the 1-5 year follow-up period.

**Conclusion:**

Rhabdomyoma are usually multiple and associated with TSC. Rhabdomyomas with TSC are larger, but most regress spontaneously or respond well to medical treatment after birth, and have an excellent long-term prognosis.

## Introduction

Fetal cardiac tumors (FCTs) are rare, and the incidence of these tumors in different series ranges from 0.08% to 0.27%. This low incidence may be related to the difficulties in ultrasonographic screening. These difficulties may include the tumor being too small or only being seen as an echogenic focus. However, with advances in non-invasive diagnostic methods, such as fetal echocardiography and magnetic resonance imaging in the last decade, the diagnosis of FCTs has become easier. Therefore, in recent years, increasing numbers of patients with FCTs have been identified prenatally (1,2).

FCTs, after excluding pericardial tumors or cysts, can be divided into two groups: benign tumors including rhabdomyomas, teratomas, fibromas, and myxomas; and malignant tumors including rhabdomyosarcomas and fibrosarcomas (3,4). FCTs, especially rhabdomyomas, are often associated with tuberous sclerosis complex (TSC). FCTs have been reported to be associated with TSC at a rate of 30-50% (5,6). Although most FCTs are benign, they may cause serious complications, such as intracardiac flow obstruction, heart valve insufficiency, rhythm disturbances, heart failure, hydrops fetalis, and even death (7). Conservative treatment or surgical resection may be a treatment option depending on tumor progression, location, number, complications, condition, and extracardiac involvement. However, the conservative approach should be prioritized unless there are severe complications in the fetus. Surgical treatment is suggested only in symptomatic patients with hemodynamically unstable FCTs or life-threatening arrhythmia.

This study aimed to evaluate the long-term follow-up of patients with FCTs who were diagnosed by fetal echocardiography in the prenatal period and to review the literature regarding advances in diagnosis and management of FCTs in the last decade.

## Material and Methods

Pregnant women who were referred to the Maternal-Fetal Medicine unit of University of Health Sciences Turkey, Kanuni Sultan Süleyman Training and Research Hospital between 2013 and 2018 for advanced ultrasonography were reviewed in this retrospective study. Data collected included the mean age of the pregnant women, parity, gestational week, gender, and the birth weight of the fetuses. Pediatric cardiology counseling was offered to all pregnant women whose fetuses had FCTs. Serial echocardiography was performed in all patients with active pregnancy management. All patients were evaluated with serial, two-dimensional, color, and pulse wave Doppler echocardiography until delivery. Echocardiography was re-performed by a pediatric cardiologist in all patients with FCTs after birth. The tumor number, location, size, and prognosis of the tumor were documented.

All patients were evaluated according to revised diagnostic criteria and also underwent genetic analysis for TSC (8). The major criteria were facial angiofibroma or forehead plaque, non-traumatic ungual or periungual fibroma, three or more hypomelanotic macules, shagreen patch (connective tissue nevus), multiple retinal nodular hamartomas, cortical tuber, subependymal nodule, subependymal giant cell astrocytoma, cardiac rhabdomyoma, lymphangiomyomatosis, and renal angiomyolipoma. Minor features were multiple, randomly distributed pits in dental enamel, hamartomatous rectal polyps, bone cysts, cerebral white matter radial migration lines, gingival fibromas, non-renal hamartomas, retinal achromic patch, “confetti” skin lesions, and multiple renal cysts. TSC was diagnosed if either two major criteria or one major criterion plus two minor features were present. TSC was also accepted in patients with positive genetic analyses. Moreover, molecular genetic testing was performed to detect *TSC1* and *TSC2* gene mutations for TSC.

Medical treatment was administered to patients with FCTs ≥30 mm or if they were symptomatic. Everolimus therapy at a dose of 2x0.25 mg twice a week for three months was started in the postnatal period and closely monitored by assessing lipid parameters and with echocardiography.

The Local Ethics Committee of University of Health Sciences Turkey, Kanuni Sultan Süleyman Training and Research Hospital approved the study (approval number: 2019/144). We obtained informed consent forms from all participants.

### Statistical analysis

The statistical analysis was performed using IBM SPSS Statistics for Windows, Version 21.0 (IBM Corp., Armonk, NY, USA). A descriptive statistical analysis was performed. Continuous variables were expressed as mean ± standard deviation or median values, and categorical variables were presented as numbers and percentages. The Kolmogorov-Smirnov test was used to evaluate the distribution of continuous variables, and a paired Samples t-test was used to compare measurements. A p-value of <0.05 was considered statistically significant.

## Results

Out of the 75,312 patients referred for advanced ultrasonographic examinations, 18 (0.024%) were diagnosed as having FCTs. The pregnant women whose fetuses had cardiac tumors were often multiparous (11 patients, 61.1%), and their mean age was 29.9±5.2 years. All patients were diagnosed during the fetal period. The median (range) gestational week at diagnosis was 28.5 (20-35) weeks. Out of the 18 patients, six were referred because of fetal arrhythmias. The others were diagnosed as having FCTs during routine ultrasonographic follow-up. None of the patients had fetal extracardiac sonographic findings. Prenatal screening for Down Syndrome was not performed in any patient due to advanced gestational week at the time of presentation. All patients were followed-up monthly during pregnancy.

Eleven (61.1%) of the fetuses diagnosed as having FCTs were male. Also, 10 patients (55.5%) with FCTs were diagnosed as having TSC. The diagnosis of TSC was made by performing a molecular genetic test after birth. Female sex (6 patients) was more common in patients with TSC-diagnosed FCTs. Two patients previously diagnosed as FCT associated with TSC also had autism. The median follow-up was 3.5 years (Table 1).

All patients were evaluated as having rhabdomyomas according to location, echogenicity (nodular hyperechogenicity), and echotexture. FCTs had multiple locations in 16 patients. 62.5% of these patients were diagnosed as having TSC. The large part of the tumors originated from the left and right ventricles (Table 2). Also, the mean tumor diameter of the fetuses with TSC was significantly larger than those without TSC (29.8±14.1 mm versus 9.3±4.8 mm, respectively, p=0.004).

All patients who received medical therapy had a diagnosis of TSC and multiple FCTs. Two patients who underwent medical treatment, a reduction in tumor size was observed. The tumor size decreased in eight patients spontaneously without a treatment during follow-up and increased in only one patient. However, there were no changes in tumor size in seven patients. In the case of one FCT with multiple locations and without a diagnosis of TSC, tumor size increased. Four patients received antiarrythmic treatment. None of the fetuses died during the follow-up period.

## Discussion

**Figure 1:**
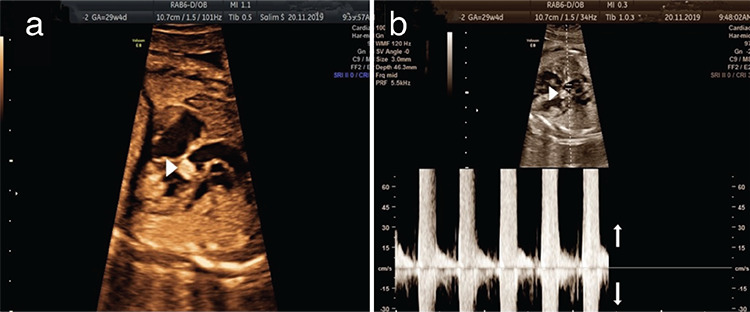
A rhabdomyoma is located at the level of the aortic valve (arrow head) (a). Power Doppler obtained just above the tumor at the beginning of the ascending aorta (b). Bidirectional flow demonstrated aortic regurgitation (arrows)

In this case series the following findings were observed: (1) the incidence of FCTs was 0.024%; (2) these tumors often had multiple locations and were often associated with TSC; (3) the tumors related to TSC were larger than those with no evidence of TSC; (4) the size of tumors was frequently reduced with medical therapy. Although FCTs are often associated with a benign prognosis, they may cause severe conditions such as hydrops fetalis and may require further intervention. Tumors, especially those located at the level of the atrio-ventricular or semilunar valves, may impair cardiac function (Figure 1).

FCTs are rare, but they may cause serious conditions such as life-threatening arrhythmias, heart failure, or death (9). Therefore, early diagnosis of these tumors is essential. The incidence of FCTs differs across series. In autopsy studies performed in all age groups, FCTs ranged between 0.0017% and 0.28% (10). In a study by Zhou et al. (11), 16,866 fetuses with a high risk of cardiac malformation were evaluated, and the incidence of FCTs was reported to be 0.08%. In our study, the incidence of FCTs was 0.024%. The reason for a lower incidence of FCTs may be the inclusion of all fetuses referred for advanced ultrasonography, not specifically for fetal echocardiography. In addition, variable inclusion criteria, environmental and genetic factors, and regional differences may be the cause for the varying incidences of FCTs.

FCTs are often benign, and malignant tumors are extremely rare. The most common of benign cardiac tumors are rhabdomyomas (60%), teratomas (25%), and fibromas (12%) (3,12). Rhabdomyoma usually presents as a nodular, hyperechogenic mass, often multiple, and is variable in size. Rhabdomyomas can be intramural or intracavitary in any cardiac chamber but often originate from the interventricular septum or right ventricle (12). In our study, all rhabdomyomas, except in two cases, were multiple and were generally located in ventricles and interventricular septa.

In a study by Lee et al. (13) there were 10 male (58.8%) and seven female (41.2%) newborns among the 17 rhabdomyoma patients. The sex distribution in our case series was very similar to this with 11 fetuses (61.1%) being male.

Rhabdomyomas are thought to be hormone-sensitive tumors, in which a decrease in dimensions is expected in the postpartum period (14). In our study, the dimensions of eight rhabdomyomas with a smaller size decreased spontaneously. However, in two patients, the tumor regressed with medical treatment. These two fetuses, who had life-threatening findings and were not suitable for surgery, were treated with everolimus, the mammalian target of the inhibitor of rapamycin. Rhabdomyomas were usually multiple and were associated with TSC.

Rhabdomyoma related to TSC is generally larger than those without TSC (15,16) as was the case in our patients.

It is widely accepted that the treatment for symptomatic cardiac tumors is surgical. However, because rhabdomyomas are multiple, localized, and infiltrative, surgical treatment is difficult and should be performed in limited cases. Although the United States Food and Drug Administration has not yet approved the treatment of cardiac rhabdomyomas with everolimus, many rhabdomyoma cases have been medically treated with this agent (17). Rhabdomyomas are benign tumors, and their long-term prognosis is excellent (7). We observed no complications due to cardiac tumors except the presence of hydrops fetalis in one fetus.

### Study limitation

The main limitation of the present study is the relatively small sample size. The patients included in our study were followed at a single center.

## Conclusion

Rhabdomyoma are usually multiple and associated with TSC. Compared with non-TSC, rhabdomyomas with TSC are larger but most regress spontaneously or respond well to medical treatment after birth. Affected babies have an excellent long-term prognosis.

## Figures and Tables

**Table 1:**
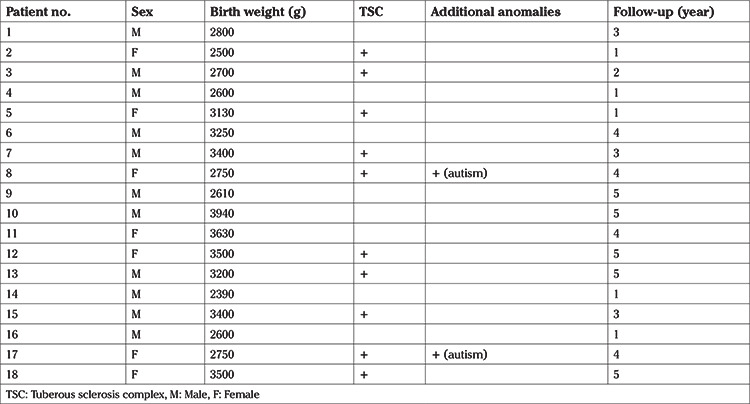
Demographic features of patients

**Table 2:**
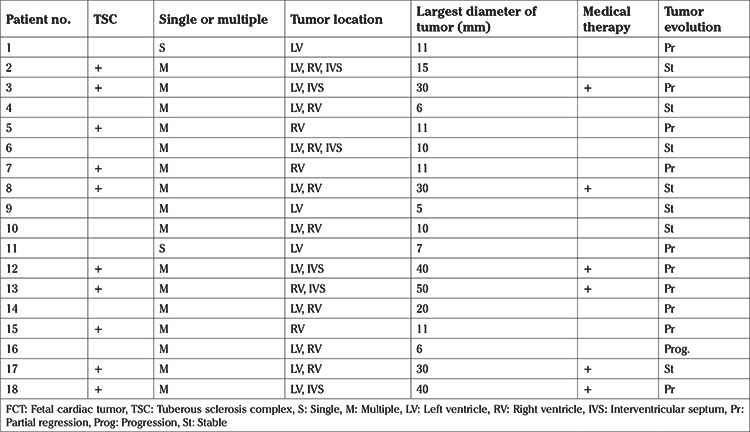
Characteristics of FTCs
